# Entropy Analysis of a Flexible Markovian Queue with Server Breakdowns

**DOI:** 10.3390/e22090979

**Published:** 2020-09-03

**Authors:** Messaoud Bounkhel, Lotfi Tadj, Ramdane Hedjar

**Affiliations:** 1 Department of Mathematics, King Saud University, Riyadh 11451, Saudi Arabia; 2 Department of Industrial Engineering, Alfaisal University, Riyadh 12714, Saudi Arabia; ltadj@alfaisal.edu; 3 Department of Computer Engineering, King Saud University, Riyadh 11453, Saudi Arabia; hedjar@ksu.edu.sa

**Keywords:** Markovian queue, flexible server, unreliable server, steady-state distribution, maximum entropy principle

## Abstract

In this paper, a versatile Markovian queueing system is considered. Given a fixed threshold level *c*, the server serves customers one a time when the queue length is less than *c*, and in batches of fixed size *c* when the queue length is greater than or equal to *c*. The server is subject to failure when serving either a single or a batch of customers. Service rates, failure rates, and repair rates, depend on whether the server is serving a single customer or a batch of customers. While the analytical method provides the initial probability vector, we use the entropy principle to obtain both the initial probability vector (for comparison) and the tail probability vector. The comparison shows the results obtained analytically and approximately are in good agreement, especially when the first two moments are used in the entropy approach.

## 1. Introduction

The concept of entropy was introduced by Shannon in his seminal papers, Shannon [1]. In information theory, entropy refers to a basic quantity associated to a random variable. Among a number of different probability distributions that express the current state of knowledge, the maximum entropy principle allows to choose the best one, that is the one with maximum entropy.

Originally, the entropy was created by Shannon as part of his theory of communication. However, since then, the principle of maximum entropy has found applications in a multitude of other areas such as statistical mechanics, statistical thermodynamics, business, marketing and elections, economics, finance, insurance, spectral analysis of time series, image reconstruction, pattern recognition, operations research, reliability theory, biology, medicine, and so forth, see Kapur [2].

In operations research, and particularly in queueing theory, a large number of papers has used the maximum entropy principle to determine the steady-state probability distribution of some process. The earliest document using entropy maximization in the context of queues that came to our attention is that of Bechtold et al. [3]. Among the latest theoretical papers applying the maximum entropy principle we cite Yen et al. [4], Shah [5], and Singh et al. [6], while She et al. [7], Giri and Roy [8], and Lin et al. [9] present recent applications.

The intention of this paper is to resume work on a paper started by Bounkhel et al. [10], who studied a flexible queueing system and used an analytical method to obtain the initial steady state probability vector. For other possible approaches to calculate the probabilities see the references in Reference [10]. The objective of this paper is threefold. First, the maximum entropy principle is used to derive the initial steady state probability vector and make sure it is in agreement with the one obtained by Bounkhel et al. [10]. Second, we use the maximum entropy principle to obtain the tail steady state probability vector. Third, improve both initial and tail probability vectors by providing more information to the maximum entropy technique.

The rest of this paper is structured as follows. In Section 2, we describe the flexible queueing system and recall the results obtained by Bounkhel et al. [10]. Our main results are presented in Section 3 where we use the maximum entropy principle to obtain the different probabilities. The theoretical results are verified with numerical illustrations. The paper is concluded in Section 4.

## 2. Model Formulation and Previous Results

Bounkhel et al. [10] studied a versatile single-server queueing system where service is regulated by an integer threshold level c≥2, and can be either single or batch as follows—if the queue length is less than *c*, then service is single and exponential with parameter $\mu_{1}$. If the queue length is equal to *c*, then service is batch of size *c* and follows the exponential distribution with parameter $\mu_{2}-\mu_{1}>0$. Finally, if the queue size is greater than *c*, then service is again batch of fixed size *c* and follows the exponential distribution with parameter $\mu_{2}$. The server is subject to breakdowns which happen according to a Poisson process with rate $\alpha_{1}$ when service is single and $\alpha_{2}$ when service is batch. Repairs that follow breakdowns are exponentially distributed with rate $\beta_{1}$ when service is single and $\beta_{2}$ when service is batch. Assume that costumers arrive according to a Poisson process with positive rate λ.

We let X(t) represent the number of customers in the system at time *t* and introduce $w_{n}$, n=0,1,2,⋯ the probability of *n* customers in the system in the steady-state when the server is in a working state, and $p_{n}$ the probability of *n* customers in the system in the steady-state regardless of the server state. Also, for |z|≤1, we introduce the probability generating functions:

$$W\left(z\right)=\sum_{n=0}^{\infty }w_{n}z^{n}\;and\;P\left(z\right)=\sum_{n=0}^{\infty }p_{n}z^{n}.$$

Then,

(1) $$\begin{array}{ccc} W(z) & = & \frac{A_{1}\left(z\right)S_{1}\left(z\right)+A_{2}\left(z\right)w_{0}z^{c-1}+A_{3}\left(z\right)z^{c}w_{1}}{z^{c}\left(\lambda +\mu_{2}\right)-\lambda z^{c+1}-\mu_{2}}, \end{array}$$

(2) $$\begin{array}{ccc} P(z) & = & \left(1+\frac{\alpha_{2}}{\beta_{2}}\right)W\left(z\right)+\left(\frac{\alpha_{1}}{\beta_{1}}-\frac{\alpha_{2}}{\beta_{2}}\right)S_{1}\left(z\right), \end{array}$$

where

$$\begin{array}{ccc} A_{1}\left(z\right):=\left(\mu_{2}-\mu_{1}\right)z^{c}+\mu_{1}z^{c-1}-\mu_{2}, & \; & A_{3}\left(z\right):=\frac{\mu_{1}^{2}\left(z^{c-1}-1\right)}{\mu_{1}-\mu_{2}},\\ A_{2}\left(z\right):=\left(\lambda +\mu_{1}\right)z-\mu_{1}+\frac{\lambda z(\mu_{1}z^{c-1}-\mu_{2})}{\mu_{2}-\mu_{1}}, & \; & S_{1}\left(z\right):=\sum_{n=0}^{c-1}w_{n}z^{n}. \end{array}$$

The *c* unknown probabilities $w_{n},n=0,1,\cdots ,c-1$, are determined by solving the system of *c* equations:

(3) $$\begin{array}{ccc} A_{1}\left(z\right)S_{1}\left(z\right)+A_{2}\left(z\right)w_{0}z^{c-1}+A_{3}\left(z\right)z^{c}w_{1}{|}_{z=z_{i}} & = & 0,\;i=1,2,\cdots ,c-1, \end{array}$$

(4) $$\begin{array}{ccc} \sum_{n=0}^{c-1}a_{n}w_{n} & = & 1, \end{array}$$

where $z_{i}$ are the c−1 roots inside the open unit ball of the equation

$$-\lambda z^{c+1}+z^{c}\left(\lambda +\mu_{2}\right)-\mu_{2}=0,$$

and

$$a_{n}=\left\{\begin{array}{cc} \frac{\left(\alpha_{2}+\beta_{2}\right)\left[A_{1}^{\prime }\left(1\right)+A_{2}^{\prime }\left(1\right)\right]}{\beta_{2}\left(c\mu_{2}-\lambda \right)}+\left(\frac{\alpha_{1}}{\beta_{1}}-\frac{\alpha_{2}}{\beta_{2}}\right), & n=0,\\ \frac{\left(\alpha_{2}+\beta_{2}\right)\left[A_{1}^{\prime }\left(1\right)+A_{3}^{\prime }\left(1\right)\right]}{\beta_{2}\left(c\mu_{2}-\lambda \right)}+\left(\frac{\alpha_{1}}{\beta_{1}}-\frac{\alpha_{2}}{\beta_{2}}\right), & n=1,\\ \frac{\left(\alpha_{2}+\beta_{2}\right)A_{1}^{\prime }\left(1\right)}{\beta_{2}\left(c\mu_{2}-\lambda \right)}+\left(\frac{\alpha_{1}}{\beta_{1}}-\frac{\alpha_{2}}{\beta_{2}}\right):=a, & n\geq 2, \end{array}\right.$$

with

$$A_{1}^{\prime }\left(1\right)=c\mu_{2}-\mu_{1},\;A_{2}^{\prime }\left(1\right)=\left(\lambda +\mu_{1}\right)+\frac{\lambda (c\mu_{1}-\mu_{2})}{\mu_{2}-\mu_{1}},\;A_{3}^{\prime }\left(1\right)=\frac{\left(c-1\right)\mu_{1}^{2}}{\mu_{1}-\mu_{2}}.$$

Writing $W\left(z\right)=\frac{N(z)}{D(z)}$, the expected number of customers in the system in the steady-state is

(5) $$E\left(X\right)=\left(1+\frac{\alpha_{2}}{\beta_{2}}\right)W^{\prime }\left(1\right)+\left(\frac{\alpha_{1}}{\beta_{1}}-\frac{\alpha_{2}}{\beta_{2}}\right)S_{1}^{\prime }\left(1\right),$$

where

$$W^{\prime }\left(1\right)=\frac{N^{\prime \prime }\left(1\right)D^{\prime }\left(1\right)-N^{\prime }\left(1\right)D^{\prime \prime }\left(1\right)}{2D^{\prime }{\left(1\right)}^{2}},$$

with

$$\begin{array}{ccc} D^{\prime }\left(1\right) & = & c\mu_{2}-\lambda ,\\ D^{\prime \prime }\left(1\right) & = & c\left[\left(c-1\right)\left(\mu_{2}+\lambda \right)-\lambda \left(c+1\right)\right],\\ N^{\prime }\left(1\right) & = & A_{1}^{\prime }\left(1\right)S_{1}\left(1\right)+A_{2}^{\prime }\left(1\right)w_{0}+A_{3}^{\prime }\left(1\right)w_{1},\\ N^{\prime \prime }\left(1\right) & = & A_{1}^{\prime \prime }\left(1\right)S_{1}\left(1\right)+2A_{1}^{\prime }\left(1\right)S_{1}^{\prime }\left(1\right)+\left[A_{2}^{\prime \prime }\left(1\right)+2\left(c-1\right)A_{2}^{\prime }\left(1\right)\right]w_{0}\\ & & +\left[A_{3}^{\prime \prime }\left(1\right)+2cA_{3}^{\prime }\left(1\right)\right]w_{1}, \end{array}$$

and

(6) $$A_{1}^{\prime \prime }\left(1\right)=\left(c-1\right)\left(c\mu_{2}-2\mu_{1}\right),\;A_{2}^{\prime \prime }\left(1\right)=\frac{\lambda \mu_{1}c\left(c-1\right)}{\mu_{2}-\mu_{1}},\;A_{3}^{\prime \prime }\left(1\right)=\left(c-2\right)A_{3}^{\prime }\left(1\right).$$

## 3. Entropy Approach

By solving the system of Equations (3)–(4), only the probabilities $w_{n},n=0,1,\cdot ,c-1$ are obtained. The rest of the probabilities $w_{n},n=c,c+1,\cdots$ can be obtained by successive differentiations of (1). Note that using (2), we have

(7) $$p_{i}=\left\{\begin{array}{cc} \left(1+\frac{\alpha_{1}}{\beta_{1}}\right)w_{i}, & i<c,\\ \left(1+\frac{\alpha_{2}}{\beta_{2}}\right)w_{i}, & i\geq c. \end{array}\right.$$

Therefore, the initial probability vector $P_{i}=\left(p_{0},p_{1},\cdots ,p_{c-1}\right)$ is completely determined while the tail probability vector $P_{t}=\left(p_{c},p_{c+1},\cdots \right)$ is yet to be determined. However, since the first moment E(X) of the process X(t) has been found in (5), we can use this information, along we the maximum entropy principle, to obtain approximate values for the components of the tail probability vector $P_{t}$.

### 3.1. Entropy Solution Using the First Moment

In a first step, we will calculate the initial probability vector using the maximum entropy principle and compare it with the initial probability vector obtained in the previous section to make sure they are in agreement. To this end, consider the following nonlinear maximization problem:

$$\begin{array}{ccc} & \max & Z=-\sum_{i=0}^{\infty }p_{i}\ln p_{i} \end{array}$$

(8) $$\begin{array}{ccc} & s.t.\; & \sum_{n=0}^{c-1}a_{n}p_{n}=1+\frac{\alpha_{1}}{\beta_{1}} \end{array}$$

(9) $$\begin{array}{ccc} \left(EP\right) & & \sum_{i=0}^{\infty }ip_{i}=E\left(X\right)\\ & & p_{i}\geq 0,\;\mathrm{for}\mathrm{all}\;i \end{array}$$

Constraint (8) is the summability-to-one condition while constraint (9) is the mean system size equation. This maximization problem can be solved by the method of Lagrange multipliers, see for example Luenberger and Ye [11]. The Lagrangian function associated with problem (EP) is given by:

$$\begin{array}{ccc} L(P_{i},\lambda ) & = & -\sum_{i=0}^{\infty }p_{i}\ln p_{i}+\lambda_{1}\left(\sum_{n=0}^{c-1}a_{n}p_{n}-1-\frac{\alpha_{1}}{\beta_{1}}\right)\\ & & +\lambda_{2}\left(\sum_{i=0}^{\infty }ip_{i}-E\left(X\right)\right), \end{array}$$

where the vector $\lambda =(\lambda_{1},\lambda_{2})$ stands for the Lagrange multipliers. Setting the derivative of $L(P_{i},\lambda )$ with respect to $p_{k}$ to zero yields

(10) $$p_{k}=e^{-1}e^{a_{k}\lambda_{1}}e^{k\lambda_{2}},\;k=0,1,2,\cdots .$$

Substituting (10) in the constraints (8) and (9), we get:

(11) $$e^{a_{0}\lambda_{1}}+e^{a_{1}\lambda_{1}}e^{\lambda_{2}}+e^{a\lambda_{1}}e^{2\lambda_{2}}\frac{1-e^{\left(c-2\right)\lambda_{2}}}{1-e^{\lambda_{2}}}+\frac{e^{c\lambda_{2}}}{1-e^{\lambda_{2}}}=e$$

(12) $$\begin{array}{c} e^{a_{1}\lambda_{1}}e^{\lambda_{2}}+e^{a\lambda_{1}}e^{3\lambda_{2}}\frac{1+\left(c-3\right)e^{\left(c-2\right)\lambda_{2}}-\left(c-2\right)e^{\left(c-3\right)\lambda_{2}}}{{\left(1-e^{\lambda_{2}}\right)}^{2}} \end{array}\;\begin{array}{c} +2e^{2\lambda_{2}}\frac{1-e^{\left(c-2\right)\lambda_{2}}}{1-e^{\lambda_{2}}}+\frac{e^{\left(c+1\right)\lambda_{2}}}{{\left(1-e^{\lambda_{2}}\right)}^{2}}+\frac{ce^{c\lambda_{2}}}{1-e^{\lambda_{2}}}=eE\left(X\right). \end{array}$$

All we need to do now is solve numerically the nonlinear system (11) and (12) to find $e^{\lambda_{1}}$ and then $e^{\lambda_{2}}$ and then substitute in (10) to obtain the probabilities $p_{k}$.

**Example** **1.** *Some numerical tests are conducted here to see how good is the solution procedure proposed in this section. In this sequel, we will refer to the solution obtained analytically as the exact solution, and to the solution obtained using the entropy approach with the first moment as the approximate solution 1. To compare these solutions, we will use the percentage error (PE1):*

(13) $$PE\;1=\left(\frac{|exact\;value-approximate\;value\;1|}{exact\;value}\right).$$

*Let us take a numerical example where c=5 and calculate the initial probability vector $P_{i}\;=\;\left(p_{0},\cdots ,p_{4}\right)$. Assume $\mu_{1}=2$, $\mu_{2}=5.5$, $\alpha_{1}=0.05$, $\alpha_{2}=0.08$, $\beta_{1}=0.07$, and $\beta_{2}=0.06$. Table 1 shows the exact solution, the approximate solution 1, and the percentage error for two different values of the arrival rate λ.*

*When λ=0.5, the average PE1 is 0.6032 and when λ=5.5, the average PE1 is 0.5432. The overall average percentage error is 0.5732, which can be greatly improved.*

### 3.2. Entropy Solution Using the Second Moment

In this subsection, we use (2) to calculate the second moment $E(X^{2})$ and we will show that the use of the second moment instead of the first moment as an extra constraint leads to an initial steady state probability vector that is also closer to the initial steady state probability vector obtained analytically. We find that the second moment is given by

(14) $$E\left(X^{2}\right)=\left(1+\frac{\alpha_{2}}{\beta_{2}}\right)W^{{}^{\prime \prime }}\left(1\right)+\left(\frac{\alpha_{1}}{\beta_{1}}-\frac{\alpha_{2}}{\beta_{2}}\right)S_{1}^{{}^{\prime \prime }}\left(1\right)+E\left(X\right),$$

where

$$W^{\prime \prime }\left(1\right)=\frac{V^{\prime \prime }\left(1\right)U^{\prime \prime \prime }\left(1\right)-U^{\prime \prime }\left(1\right)V^{\prime \prime \prime }\left(1\right)}{3V^{\prime \prime }{\left(1\right)}^{2}},$$

with

$$\begin{array}{ccc} V^{\prime \prime }\left(1\right) & = & 2{\left(c\mu_{2}-\lambda \right)}^{2}\\ U^{\prime \prime }\left(1\right) & = & D^{\prime }\left(1\right)N^{\prime \prime }\left(1\right)-N^{\prime }\left(1\right)D^{\prime \prime \prime }\left(1\right)\\ V^{\prime \prime \prime }\left(1\right) & = & 6c\left(c\mu_{2}-\lambda \right)\left[\left(c-1\right)\mu_{2}-\lambda \right]\\ U^{\prime \prime \prime }\left(1\right) & = & 2[D^{\prime }\left(1\right)N^{\prime \prime \prime }\left(1\right)-N^{\prime }\left(1\right)D^{\prime \prime \prime }\left(1\right)]\\ A_{1}^{\prime \prime \prime }\left(1\right) & = & \left(c-1\right)\left(c-2\right)\left(c\mu_{2}-3\mu_{1}\right)\\ A_{2}^{\prime \prime \prime }\left(1\right) & = & \frac{\lambda c(c-1)(c-2)}{\mu_{2}-\mu_{1}}\\ A_{3}^{\prime \prime \prime }\left(1\right) & = & \frac{\mu_{1}^{2}\left(c-1\right)\left(c-2\right)\left(c-3\right)}{\mu_{1}-\mu_{2}}\\ D^{\prime \prime \prime }\left(1\right) & = & c\left(c-1\right)\left[\left(c-2\right)\mu_{2}-3\lambda \right]\\ N^{\prime \prime \prime }\left(1\right) & = & 3A_{1}^{\prime \prime }\left(1\right)S_{1}^{\prime }\left(1\right)+3A_{1}^{\prime }\left(1\right)S_{1}^{\prime \prime }\left(1\right)+S_{1}\left(1\right)A_{1}^{\prime \prime \prime }\left(1\right)\\ & & +w_{0}\left[A_{2}^{\prime \prime \prime }\left(1\right)+3\left(c-1\right)A_{2}^{\prime \prime }\left(1\right)+3\left(c-1\right)\left(c-2\right)A_{2}^{\prime }\left(1\right)\right]\\ & & +w_{1}\left[A_{3}^{\prime \prime \prime }\left(1\right)+3cA_{3}^{\prime \prime }\left(1\right)+3c\left(c-1\right)A_{3}^{\prime }\left(1\right)\right]\\ S_{1}^{\prime \prime }\left(1\right) & = & \sum_{n=0}^{c-1}n\left(n-1\right)w_{n}. \end{array}$$

The nonlinear maximization problem to solve in this case is the following:

$$\begin{array}{ccc} & \max & Z=-\sum_{i=0}^{\infty }p_{i}\ln p_{i} \end{array}$$

(15) $$\begin{array}{ccc} & s.t.\; & \sum_{n=0}^{c-1}a_{n}p_{n}=1+\frac{\alpha_{1}}{\beta_{1}} \end{array}$$

(16) $$\begin{array}{ccc} \left(EP2\right) & & \sum_{i=0}^{\infty }i^{2}p_{i}=E\left(X^{2}\right)\\ & & p_{i}\geq 0,\;\mathrm{for}\mathrm{all}\;i. \end{array}$$

Similarly to the case where we only used the first moment, we use the classical method of Lagrange. The following system of nonlinear equations, where the unknowns are the Lagrange multipliers $\left(\lambda_{1},\lambda_{2}\right)$ is obtained:

$$\begin{array}{ccc} e^{a_{0}\lambda_{1}}+e^{a_{1}\lambda_{1}+\lambda_{2}}+e^{a\lambda_{1}}\sum_{k=2}^{c-1}e^{k^{2}\lambda_{2}}+\sum_{k=c}^{\infty }e^{k^{2}\lambda_{2}}-e & = & 0\\ e^{a_{1}\lambda_{1}+\lambda_{2}}+e^{a\lambda_{1}}\sum_{k=2}^{c-1}k^{2}e^{k^{2}\lambda_{2}}+\sum_{k=c}^{\infty }k^{2}e^{k^{2}\lambda_{2}}-eE\left(X^{2}\right) & = & 0 \end{array}$$

This system can be solved numerically. Once we have the values of $\left(\lambda_{1},\lambda_{2}\right)$, we replace these values in the following formula to obtain the probabilities $p_{k}$:

(17) $$p_{k}=e^{-1}e^{a_{k}\lambda_{1}}e^{k^{2}\lambda_{2}},\;k=0,1,2,\cdots .$$

**Example** **2.** *Some numerical tests are conducted here to see how good is the solution procedure proposed in this subsection. Similarly to the previous subsection, we will refer to the percentage error obtained using the entropy approach with the second moment as PE2. Then we compare the two approximate solutions using the percentage errors. Let us take a numerical example with the same data in Example 1, that is, c=5, $\mu_{1}=2$, $\mu_{2}=5.5$, $\alpha_{1}=0.05$, $\alpha_{2}=0.08$, $\beta_{1}=0.07$, and $\beta_{2}=0.06$. Table 2 shows the exact solution, the approximate solution obtained in Section 3.1, the approximate solution obtained in this subsection, the percentage errors PE1 and PE2, for two different values of the arrival rate λ.*

*When λ=0.5, the average of PE1 is 0.6032 and the average of PE2 is 0.5119, and when λ=5.5, the average of PE1 is 0.5432 and the average of PE2 is 0.4661. The overall average percentage error of PE1 and PE2, respectively, are 0.5732 and 0.4890, which can be greatly improved in the next subsection.*

### 3.3. Entropy Solution Using Both First and Second Moments

Our objective here is to improve the probability vector obtained in the previous subsections. This is realized by including both first and second moments to the previous formulation. We will show that the use of the two moments as extra constraints leads to best approximation to the initial steady state probability vector obtained analytically. The nonlinear maximization problem to solve in this case is the following:

$$\begin{array}{ccc} & \max & Z=-\sum_{i=0}^{\infty }p_{i}\ln p_{i} \end{array}$$

(18) $$\begin{array}{ccc} & s.t.\; & \sum_{n=0}^{c-1}a_{n}p_{n}=1+\frac{\alpha_{1}}{\beta_{1}} \end{array}$$

(19) $$\begin{array}{ccc} \left(EP3\right) & & \sum_{i=0}^{\infty }ip_{i}=E\left(X\right) \end{array}$$

(20) $$\begin{array}{ccc} & & \sum_{i=0}^{\infty }i^{2}p_{i}=E\left(X^{2}\right)\\ & & p_{i}\geq 0,\;\mathrm{for}\mathrm{all}\;i. \end{array}$$

Similarly to the previous cases, we use the classical method of Lagrange. The following system of nonlinear equations, where the unknowns are the Lagrange multipliers $\left(\lambda_{1},\lambda_{2},\lambda_{3}\right)$ is obtained:

$$\begin{array}{ccc} e^{a_{0}\lambda_{1}}+e^{a_{1}\lambda_{1}+\lambda_{2}+\lambda_{3}}+e^{a\lambda_{1}}\sum_{k=2}^{c-1}e^{a\lambda_{1}+k\lambda_{2}+k^{2}\lambda_{3}}+\sum_{k=c}^{\infty }e^{k\lambda_{2}+k^{2}\lambda_{3}}-e & = & 0\\ e^{a_{1}\lambda_{1}+\lambda_{2}+\lambda_{3}}+e^{a\lambda_{1}}\sum_{k=2}^{c-1}ke^{a\lambda_{1}+k\lambda_{2}+k^{2}\lambda_{3}}+\sum_{k=c}^{\infty }ke^{k\lambda_{2}+k^{2}\lambda_{3}}-eE\left(X\right) & = & 0\\ e^{a_{1}\lambda_{1}+\lambda_{2}+\lambda_{3}}+e^{a\lambda_{1}}\sum_{k=2}^{c-1}k^{2}e^{a\lambda_{1}+k\lambda_{2}+k^{2}\lambda_{3}}+\sum_{k=c}^{\infty }k^{2}e^{k\lambda_{2}+k^{2}\lambda_{3}}-eE\left(X^{2}\right) & = & 0\\ . \end{array}$$

This system can be solved numerically. The values of $\left(\lambda_{1},\lambda_{2},\lambda_{3}\right)$ obtained numerically will be replaced in the following formula to obtain the probabilities $p_{k}$:

(21) $$p_{k}=e^{-1}e^{a_{k}\lambda_{1}}e^{k\lambda_{2}}e^{k^{2}\lambda_{3}},\;k=0,1,2,\cdots .$$

**Example** **3.** *Let us take the same data as in Examples 1 and 2 and calculate the initial probability vector using the analytical method (exact), entropy approach with first moment only (Entropy 1), entropy approach with second moment only (Entropy 2), and entropy approach with both first and second moments (Entropy 1&2). Table 3 and Table 4 show the exact solution and the approximate solutions along with the corresponding percentage errors for λ=0.5 and λ=5.5, respectively.*

**Figure 1:**
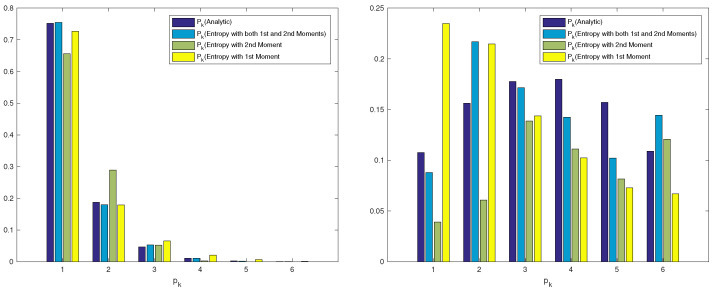
Initial probability vectors comparison (left λ=0.5 and right λ=5.5).

*We denote by PE1 the percentage error when Entropy 1 is used, by PE2 the percentage error when Entropy 2 is used, and by PE1&2 the percentage error when Entropy 1&2 is used. The overall average percentage error using the entropy approach with the first moment is 0.5732, while the overall average percentage error using the entropy approach with the second moment is 0.4890 which represents a slight improvement of $\frac{\left|0.5732-0.4890\right|}{0.5732}=14.68\%$. However, the overall average percentage using both moments is 0.1622, which represents a substantial improvement of $\frac{\left|0.5732-0.1622\right|}{0.5732}=71.70\%$. We show in Figure 1 the two distributions for a better visualisation. We can see that the entropy approach with both moments always outperforms the entropy approach with only the first moment or only second moment.*

**Figure 2:**
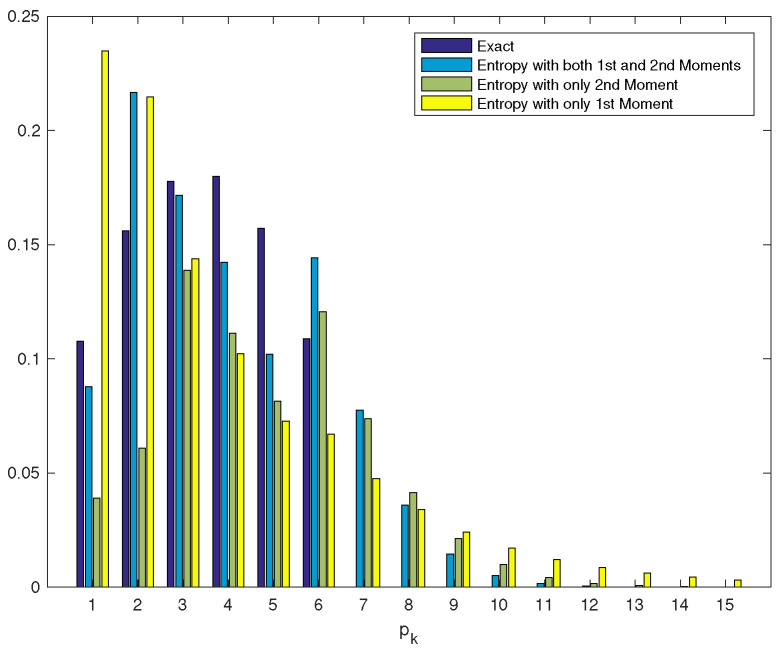
Initial and tail probability vectors.

*Since the results obtained by the entropy method with both two moments are satisfactory, we also calculated the tail probability vector and present in Figure 2 both initial and tail probability vectors. For comparison, we present the distribution obtained when only one moment (first or second moment) is used and when both first and second moments are used.*

*One other remark we make when looking at Table 2, Table 3 and Table 4 is that the probability mass function is concentrated at $p_{0}$ for small values of λ and as λ increases, this distribution becomes more evenly distributed and the value of $p_{0}$ decreases. Intuitively, this makes sense since we expect the probability of no customers in the system to decrease as the arrival rate increases. Therefore, to further compare the approximate entropy approaches, we conduct next a sensitivity analysis to investigate the effect of λ on the percentage errors of $p_{0}$. We also explore the effect of other parameters, namely c, $\mu_{1}$ and $\mu_{2}$. The parameters $\alpha_{i}$ and $\beta_{i}$ do not seem to have any effect on the deviations. For the sensitivity analysis, we keep the base values of Example 1 and change one parameter at a time.*

**Effect of λ on the Percentage Error of $p_{0}$.**

**Figure 3:**
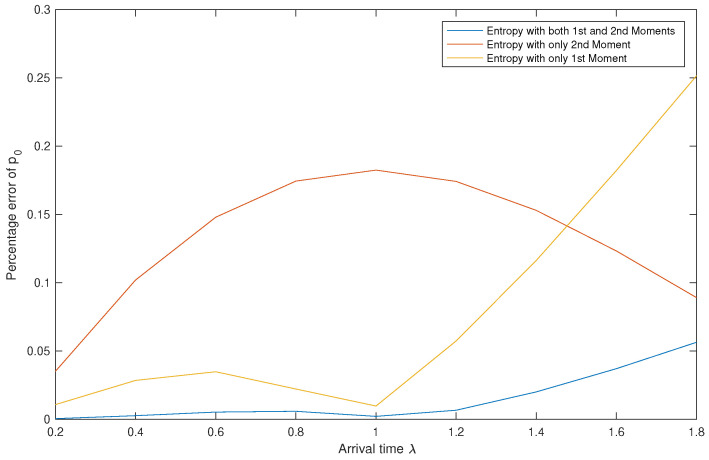
Effect of λ on the percentage errors of $p_{0}$.

*Table 5 shows the values of $p_{0}$ calculated using the three methods, while Figure 3 shows the variations of the percentage errors as λ changes.*

*We read from Table 5 two points: First, the approximation results obtained using Entropy 1&2 are clearly better than the ones obtained by the two other methods. Second, the efficiency of the best method is inversely proportional to the values of λ.*

*Figure 3 shows that Entropy 1&2 always has the lowest PE, however, there are values of λ for which PE1<PE2. In other words, if we are using a single moment, then better use the first moment for small values of λ and the second moment for larger values of λ.*

**Effect of $\mu_{1}$ on the Percentage Error of $p_{0}$.**

**Figure 4:**
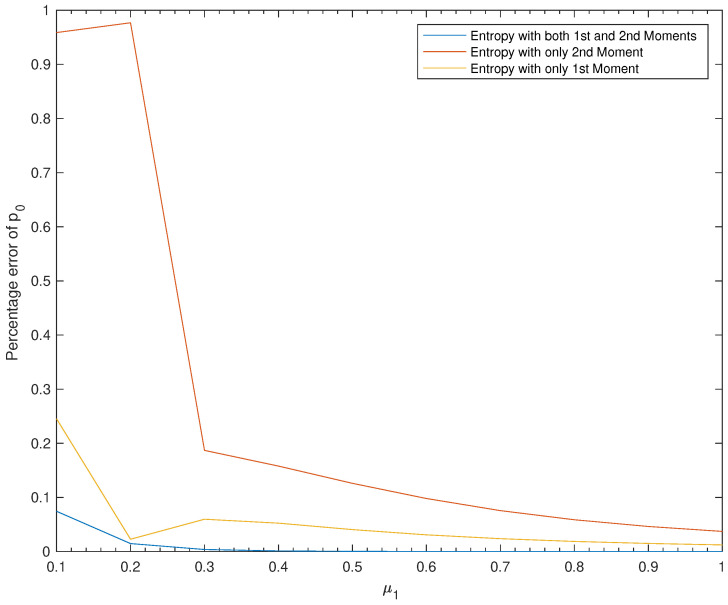
Effect of $\mu_{1}$ on the percentage errors of $p_{0}$.

*The results are summarized in Table 6 and Figure 4.*

*We read from Table 6 three points: First, the approximation results obtained using Entropy 1&2 are clearly better than the ones obtained by the two other methods. Second, Entropy 1 is much better than Entropy 2, that is, if we are using a single moment, then better use the first moment than the second moment. Third, the efficiency of all three methods is directly proportional to the values of $\mu_{1}$.*

*We can see from Figure 4 that we always have PE1&2<PE1<PE2, which confirms our conclusions from Table 6 stated above.*

**Effect of $\mu_{2}$ on the Percentage Error of $p_{0}$.**

**Figure 5:**
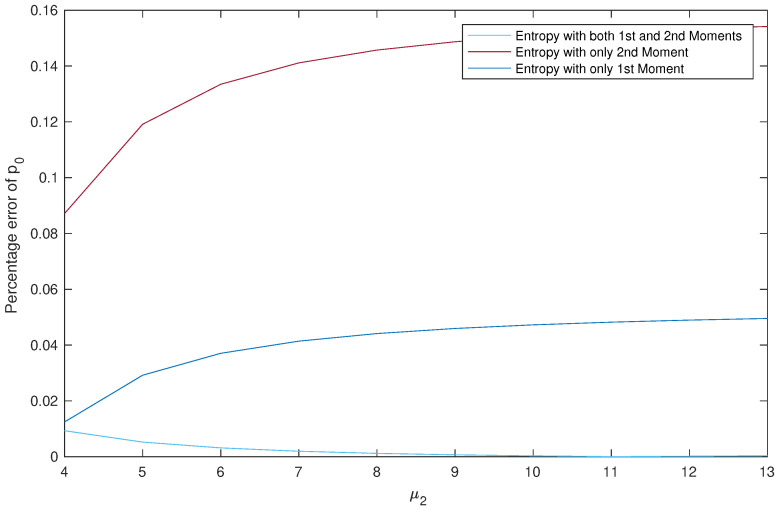
Effect of $\mu_{2}$ on the percentage errors of $p_{0}$.

*The results are summarized in Table 7 and Figure 5.*

*We read from Table 7 three points: First, the approximation results obtained using Entropy 1&2 are much better than the ones obtained by the two other methods. Second, if we are using a single moment, then better use Entropy 1 than Entropy 2. Third, the efficiency of the best method is directly proportional to the values of $\mu_{2}$ and the efficiency of the other two methods is inversely proportional.*

*Again observe from Figure 5 that we always have PE1&2<PE1<PE2, which confirms our conclusions from Table 7 stated above.*

**Effect of c on The Initial Probability Vector $P_{i}$.**

**Figure 6:**
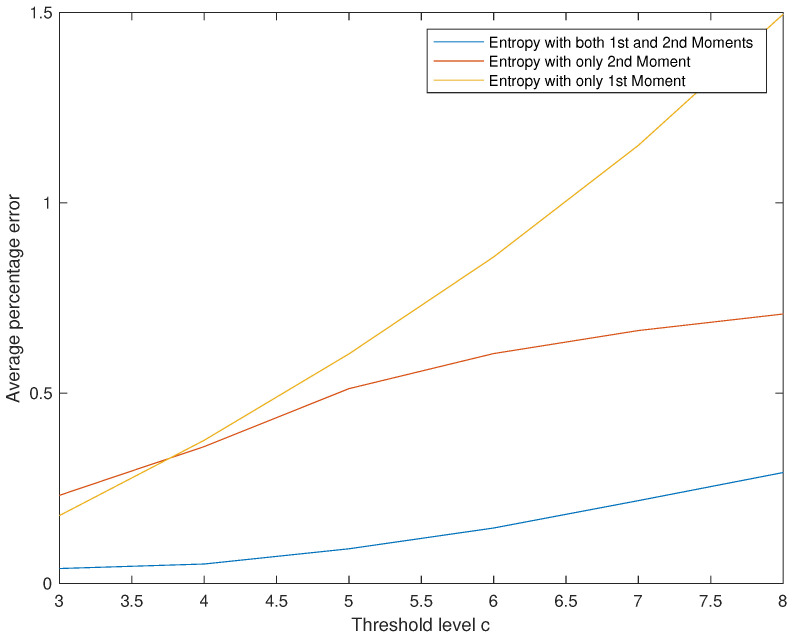
Effect of c on the average percentage error.

*The results are summarized in Table 8 and Figure 6. Superiority of Entropy 1&2 is demonstrated for all values of c.*

*We read from Table 8 and Figure 6 three points: First, obviously the approximation results obtained using Entropy 1&2 are clearly better than the ones obtained by the two other methods. Second, if we are using a single moment, then better use Entropy 2 than Entropy 1 for large values of c and for small values of c there is no big difference between the two methods. Third, the efficiency of all three methods is inversely proportional to the values of c.*

*From our previous sensitivity analysis, we conclude that if a single moment is used to estimate the probabilities, then it makes a difference whether we use the first moment or the second moment. Also, the more information we feed the maximum entropy technique, the more accurate the results are. Although we did not do it, we conjecture that inclusion of the third moment would confirm our findings that including more information would result in higher accuracy.*

## 4. Conclusions

An analytical and the maximum entropy principle are used in this paper to calculate the steady-state initial probabilities of the number of customers in a Markovian queueing system. The entropy solution is further improved by including second moment information. When the analytical and entropy solutions are in agreement, the entropy solution is used to obtain the tail probabilities of the number of customers in the system. These probabilities cannot be obtained analytically.

The number of customers in the system is a discrete random variable. This paper can be followed by one where a continuous random variable such as the waiting time or the busy period is studied. In this case, the probability density function, instead of the probability mass function, needs to be calculated.

## Figures and Tables

**Table 1:** Initial probability vectors comparison.

c=5						
λ	λ=0.5			λ=5.5		
	**Exact**	**Approx. 1**	PE1	**Exact**	**Approx. 1**	PE1
$p_{0}$	0.7519	0.7265	0.0338	0.1077	0.2349	1.1815
$p_{1}$	0.1876	0.1794	0.0441	0.1561	0.2147	0.3748
$p_{2}$	0.0465	0.0653	0.4049	0.1776	0.1438	0.1906
$p_{3}$	0.0112	0.0206	0.8346	0.1799	0.1023	0.4316
$p_{4}$	0.0024	0.0065	1.6986	0.1572	0.0727	0.5373
Average			0.6032			0.5432

**Table 2:** Initial probability vectors comparison.

c=5										
λ	λ=0.5					λ=5.5				
	**Exact**	**Appr. 1**	PE1	**Appr. 2**	PE2	**Exact**	**Appr. 1**	PE1	**Appr. 2**	PE2
$p_{0}$	0.7519	0.7265	0.0338	0.6560	0.1275	0.1077	0.2349	1.1815	0.0391	0.6372
$p_{1}$	0.1876	0.1794	0.0441	0.2892	0.5414	0.1561	0.2147	0.3748	0.0608	0.6108
$p_{2}$	0.0465	0.0653	0.4049	0.0523	0.1237	0.1776	0.1438	0.1906	0.1387	0.2191
$p_{3}$	0.0112	0.0206	0.8346	0.0025	0.7810	0.1799	0.1023	0.4316	0.1112	0.3821
$p_{4}$	0.0024	0.0065	1.6986	0.0000	0.9858	0.1572	0.0727	0.5373	0.0815	0.4812
Average			0.6032		0.5119			0.5432		0.4661

**Table 3:** Initial probability vectors comparison (λ=0.5).

	Exact	Entropy 1	PE1	Entropy 2	PE2	Entropy 1&2	PE1&2
$p_{0}$	0.7519	0.7265	0.0338	0.6560	0.1275	0.7550	0.0040
$p_{1}$	0.1876	0.1794	0.0441	0.2892	0.5414	0.1795	0.0431
$p_{2}$	0.0465	0.0653	0.4049	0.0523	0.1237	0.0527	0.1324
$p_{3}$	0.0112	0.0206	0.8346	0.0025	0.7810	0.0108	0.0346
$p_{4}$	0.0024	0.0065	1.6986	0.0000	0.9858	0.0018	0.2421
Average			0.6032		0.5119		0.0912

**Table 4:** Initial probability vectors comparison (λ=5.5).

	Exact	Entropy 1	PE1	Entropy 2	PE2	Entropy 1&2	PE1&2
$p_{0}$	0.1077	0.2349	1.1815	0.0391	0.6372	0.0878	0.1843
$p_{1}$	0.1561	0.2147	0.3748	0.0608	0.6108	0.2167	0.3881
$p_{2}$	0.1776	0.1438	0.1906	0.1387	0.2191	0.1716	0.0341
$p_{3}$	0.1799	0.1023	0.4316	0.1112	0.3821	0.1423	0.2090
$p_{4}$	0.1572	0.0727	0.5373	0.0815	0.4812	0.1021	0.3503
Average			0.5432		0.4661		0.2332

**Table 5:** Effect of λ on $p_{0}$.

λ	Exact	Entropy 1	Entropy 2	Entropy 1&2
0.2	0.5341	0.5393	0.4366	0.5352
0.4	0.2894	0.3826	0.2739	0.2673
0.6	0.1937	0.3189	0.1279	0.1618
0.8	0.1468	0.2794	0.1256	0.1169
1.0	0.1183	0.2486	0.0111	0.0949
1.2	0.0986	0.2219	0.1435	0.0821
1.4	0.0838	0.1974	0.1330	0.0733
1.6	0.0721	0.1744	0.0880	0.0660
1.8	0.0627	0.1523	0.0339	0.0597

**Table 6:** Effect of $\mu_{1}$ on $p_{0}$.

$\mu_{1}$	Exact	Entropy 1	Entropy 2	Entropy 1&2
0.1	0.3312	0.4124	0.0136	0.3065
0.2	0.5429	0.5306	0.0126	0.5349
0.3	0.6756	0.6353	0.5493	0.6730
0.4	0.7526	0.7131	0.6338	0.7517
0.5	0.8009	0.7685	0.7000	0.8007
0.6	0.8337	0.8080	0.7520	0.8337
0.7	0.8573	0.8369	0.7925	0.8574
0.8	0.8751	0.8587	0.8237	0.8752
0.9	0.8889	0.8756	0.8478	0.8890
1	0.9000	0.8890	0.8665	0.9001

**Table 7:** Effect of $\mu_{2}$ on $p_{0}$.

$\mu_{2}$	Exact	Entropy 1	Entropy 2	Entropy 1&2
4	0.7516	0.7422	0.6861	0.7586
5	0.7518	0.7299	0.6623	0.7558
6	0.7520	0.7241	0.6516	0.7544
7	0.7521	0.7210	0.6460	0.7536
8	0.7522	0.7190	0.6426	0.7531
9	0.7523	0.7177	0.6404	0.7528
10	0.7523	0.7168	0.6389	0.7525
11	0.7523	0.7161	0.6378	0.7524
12	0.7524	0.7155	0.6370	0.7523
13	0.7524	0.7151	0.6364	0.7522

**Table 8:** Effect of c on initial probability vector $P_{i}$.

c=3	$p_{0}$	$p_{1}$	$p_{2}$						
Exact	0.7687	0.1863	0.0400						
Entropy 1	0.7582	0.1707	0.0575						
Entropy 2	0.7052	0.2625	0.0318						
Entropy 1&2	0.7701	0.1824	0.0438						
c=4	$p_{0}$	$p_{1}$	$p_{2}$	$p_{3}$					
Exact	0.7562	0.1876	0.0453	0.0097					
Entropy 1	0.7348	0.1773	0.0633	0.0197					
Entropy 2	0.6723	0.2810	0.0449	0.0017					
Entropy 1&2	0.7582	0.1819	0.0500	0.0091					
c=5	$p_{0}$	$p_{1}$	$p_{2}$	$p_{3}$	$p_{4}$				
Exact	0.7519	0.1876	0.0465	0.0112	0.0024				
Entropy 1	0.7265	0.1794	0.0653	0.0206	0.0065				
Entropy 2	0.6560	0.2892	0.0523	0.0025	0.0000				
Entropy 1&2	0.7550	0.1795	0.0527	0.0108	0.0018				
c=6	$p_{0}$	$p_{1}$	$p_{2}$	$p_{3}$	$p_{4}$	$p_{5}$			
Exact	0.7506	0.1876	0.0468	0.0116	0.0028	0.0006			
Entropy 1	0.7236	0.1801	0.0662	0.0209	0.0066	0.0021			
Entropy 2	0.6488	0.2927	0.0557	0.0028	0.0000	0.0000			
Entropy 1&2	0.7544	0.1777	0.0536	0.0117	0.0022	0.0003			
c=7	$p_{0}$	$p_{1}$	$p_{2}$	$p_{3}$	$p_{4}$	$p_{5}$	$p_{6}$		
Exact	0.7502	0.1875	0.0469	0.0117	0.0029	0.0007	0.0001		
Entropy 1	0.7224	0.1804	0.0665	0.0211	0.0067	0.0021	0.0007		
Entropy 2	0.6457	0.2941	0.0571	0.0030	0.0000	0.0000	0.0000		
Entropy 1&2	0.7545	0.1766	0.0540	0.0121	0.0023	0.0004	0.0001		
c=8	$p_{0}$	$p_{1}$	$p_{2}$	$p_{3}$	$p_{4}$	$p_{5}$	$p_{6}$	$p_{7}$	
Exact	0.7500	0.1875	0.0469	0.0117	0.0029	0.0007	0.0002	0.0000	
Entropy 1	0.7219	0.1805	0.0668	0.0211	0.0067	0.0021	0.0007	0.0002	
Entropy 2	0.6445	0.2947	0.0577	0.0031	0.0001	0.0000	0.0000	0.0000	
Entropy 1&2	0.7547	0.1761	0.0541	0.0122	0.0024	0.0004	0.0001	0.0000	
